# Application of loop analysis for the qualitative assessment of surveillance and control in veterinary epidemiology

**DOI:** 10.1186/1742-7622-10-7

**Published:** 2013-08-13

**Authors:** Lucie Collineau, Raphaël Duboz, Mathilde Paul, Marisa Peyre, Flavie Goutard, Sinel Holl, François Roger

**Affiliations:** 1 CIRAD, AGIRs (Animal and Integrated Risks Management Unit, UPR22), Montpellier, France; 2 UMR 1225 IHAP INRA-ENVT, National Veterinary School, Toulouse, France; 3 National Veterinary Research Institute, Phnom Penh, Cambodia; 4 Asian Institute of Technology, Computer Sciences and Information Management, Pathumthani, Thailand; 5 Faculty of Veterinary Medicine, Kasetsart University, Bangkok, Thailand

**Keywords:** Efficiency, Mitigation system, Loop analysis, Interaction, HPAI H5N1

## Abstract

**Background:**

Systems for animal disease mitigation involve both surveillance activities and interventions to control the disease. They are complex organizations that are described by partial or imprecise data, making it difficult to evaluate them or make decisions to improve them. A mathematical method, called loop analysis, can be used to model qualitatively the structure and the behavior of dynamic systems; it relies on the study of the sign of the interactions between the components of the system. This method, currently widely used by ecologists, has to our knowledge never been applied in the context of animal disease mitigation systems. The objective of the study was to assess whether loop analysis could be applied to this new context. We first developed a generic model that restricted the applicability of the method to event-based surveillance systems of endemic diseases, excluding the emergence and eradication phases. Then we chose the mitigation system of highly pathogenic avian influenza (HPAI) H5N1 in Cambodia as an example of such system to study the application of loop analysis to a real disease mitigation system.

**Results:**

Breaking down the generic model, we constructed a 6-variables model to represent the HPAI H5N1 mitigation system in Cambodia. This construction work improved our understanding of this system, highlighting the link between surveillance and control which is unclear in traditional representations of this system. Then we analyzed the effect of the perturbations to this HPAI H5N1 mitigation system that we interpreted in terms of investment in a given compartment. This study suggested that increasing intervention at a local level can optimize the system’s efficiency. Indeed, this perturbation both decreases surveillance and intervention costs and reduces the disease’s occurrence.

**Conclusion:**

Loop analysis can be applied to disease mitigation systems. Its main strength is that it is easy to design, focusing on the signs of the interactions. It is a simple and flexible tool that could be used as a precursor to large-scale quantitative studies, to support reflection about disease mitigation systems structure and functioning.

## Background

### The context of animal disease mitigation systems

An animal disease surveillance system consists of all individuals or agencies organized to ensure surveillance in a given region of one or more hazards [1]. Surveillance consists of the continuous collection, recording and analysis of data that are then disseminated in order to control the disease [2], i.e. to reduce the disease prevalence, to early detect cases or to demonstrate the freedom from infection or disease. An intervention aims to control the disease. As suggested by Häsler et al., surveillance and intervention activities are part of a mitigation strategy to make the effect of the disease less severe [3]. The term disease mitigation system is further used in the text as a synonym of disease surveillance and control systems. The activities of surveillance and intervention vary according to the disease being studied and the objectives of the system. Vaccination, culling (test-and-slaughter), vector control or biosecurity improvement are measures that can be applied to control an animal disease. These activities vary in terms of their cost (economic and social), their effectiveness and their feasibility [4,5].

Disease mitigation systems are complex organizations, and the data they collect are partial, incomplete or imprecise, particularly in developing countries [6]. The evaluation of surveillance programs is crucial to i) assess the limitations and usefulness of the data generated [7], ii) ensure that limited resources are effectively used for protecting health [8] and iii) comply with requirements of international organizations in the era of trade globalization [9]. Different methods and tools exist for the qualitative and quantitative evaluation of animal disease surveillance systems: e.g. OASIS (Outil d’analyse de système d’information en santé) tool [7]; SERVAL (SuRveillance EVALuation framework) [10]; tree scenarios [11], or capture-recapture techniques [12]. Current representations of animal surveillance systems are mostly organigrammes or flowcharts. They provide only an organizational snapshot of these systems, and a limited understanding of their process dynamics (in particular the link between surveillance and intervention activities). This hinders both the evaluation of these systems and any decision on how to improve their efficiency, defined as how effective they are in reducing the prevalence of the disease by making the best use of the financial, human and material resources they mobilize [13].

### The method of loop analysis

Feedback loop analysis, referred to simply as loop analysis is a method that applies qualitative models to dynamic systems [14]. It allows i) to represent the system’s components and interactions between components, and ii) from the study of the sign of the interactions, to predict the response of the system to the modification of the level of one of its variable; this modification is called perturbation. While the loop analysis method is frequently applied in ecology [15,16] and has been used for exploring associations between various variables and epidemiological risks [17,18], it has to our knowledge never been used to study animal disease mitigation systems. In this section, we first present the approach one has to follow to conduct loop analysis.

Let us consider a dynamic system composed of n continuous variables. For i = 1 to n, X_i_ represents the i^th^ system’s component. Loop analysis is conducted following seven successive steps:

1) First, the system’s components (or variables) are described.

2) Loop analysis requires making the following hypothesis: i) the system is close to a state of equilibrium at every moment and ii) interactions are considered continuous, monotone and of the same order of magnitude.

3) The interactions between the system’s variables are defined and represented by a signed directed graph, i.e. a graph in which the arrows representing interactions have a given direction and sign (positive, negative or no interaction from one variable to another or to itself).

4) The directed graph is converted into a unique interaction matrix labelled A, in which the elements a_ij_ represent the effect of a modification of X_j_ on X_i_. The value of elements a_ij_ can be any of {+1; -1; 0}, depending on whether the effect of modifying X_j_ on X_i_ is positive, negative, or null, respectively. To get the interaction matrix from the directed graph, only the direction and the sign of the interactions are taken into account, and not its amplitude.

5) We verify that the system meets both the Hurwitz criteria for stability, and is therefore stable. These criteria, based on matrix determinants calculations, mean that the system always returns to its equilibrium after a perturbation. For further details about the calculation method, please see the work from Dambacher et al. [19].

6) We study the response of the system to perturbations to its different variables, by calculating the conjugate transpose (also called adjoint matrix) labelled Adj(-A) for interaction matrix A (see [20] for calculation details). The signs of the adjoint matrix coefficients give the direction of change (increase or decrease) of all the system’s variables after the increase (respectively decrease) of the level of one variable of the system (called positive, respectively negative perturbation of this variable). Perturbations are represented in the columns and variables’ responses in the rows of the matrix Adj(-A). The response of the system to perturbations can also be displayed using a directed graph in which decreasing variables are represented by small size circles, increasing variables by big size circles and variables having undergone no effect or ambiguous effect by an intermediate size circle.

7) We calculate the matrix W of weighted predictions which allows for a level of confidence to be attributed to the predictions [20], ranging from unambiguous at level w_ij_ = 1 (certain prediction), to totally ambiguous at w_ij_ = 0 (very poor confidence in the prediction). A value of w_ij_ = 0.5 is generally accepted as a validation threshold.

Loop analysis is a dynamic system modeling method. To our knowledge, dynamic modeling has been little used to study disease mitigation systems. An example is provided by Howe et al. who developed a generic model to study the overall economic optimum for disease mitigation [21]. In the wider context of epidemiology, dynamic modeling has been mainly applied to represent the spread of diseases [22]. For example, SIR model is a compartmental model representing the dynamic of the disease between three compartments (or states): susceptible, infectious and recovered. Compartmental models can also be used to study the impact of certain control measures (e.g. vaccination) on the dynamic of infection. The main difference with loop analysis is that time is considered (e.g. we observe the moment at which the outbreak goes extinct). Another difference is that compartmental models require quantifying the interactions between compartments, which is sometimes difficult or impossible; that is not necessary with loop analysis which only requires signs to be defined. This is the strength of loop analysis (easy to design), but also its main limit (the model only predicts whether variables increase or decrease, but not how much and how fast). In addition, loop analysis allows the integration of variables of very different natures, which in turn enables the development of interdisciplinary approaches [23]. The whole approach can also be computerized using for example the Loop analysis website [24].

The objective of this study was to assess whether loop analysis could be applied to the context of animal disease mitigation systems. More especially, we evaluated whether loop analysis allows i) to model the components of a mitigation system and the interactions between them, and ii) to study the mitigation system’s behavior after a modification of the level of one of its components (perturbation), that we interpreted in terms of system’s efficiency. We first applied loop analysis to a generic disease mitigation system. This study, presented in the next section, allowed both to determine the limits of the method applicability and the conditions necessary for the application of loop analysis to a disease mitigation system. It also provided a starting point for the construction of more complex models. Breaking down this generic model, we applied loop analysis to a real mitigation system: the HPAI H5N1 mitigation system in Cambodia. This work is described in the results section of this paper.

### Application of loop analysis to a generic disease mitigation system

We considered disease mitigation systems as dynamic systems. Following the 7-steps approach described in the previous section, we applied loop analysis to a simplified and generic model of an animal disease mitigation system, unconnected to any particular disease.

**Figure 1:**
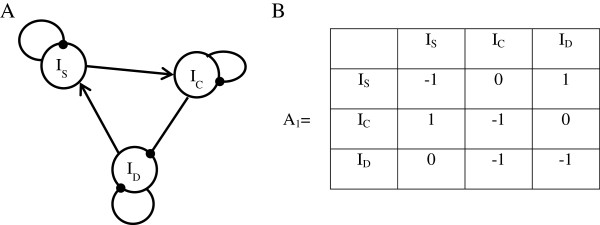
**Representation of the generic system.** I_S_: Event-based surveillance intensity. I_C_: Control intensity. I_D_: Detected disease intensity. **A**: Directed graph. Pointed end arrows: positive interaction. Circular end arrows: negative interaction. **B**: Matrix A_1_ of interactions. **A** and **B** are two equivalent representations of the 3I system. To get the interaction matrix A_1_ from the directed graph, only the direction and the sign of the interactions are taken into account, and not their amplitude.

**Figure 2:**
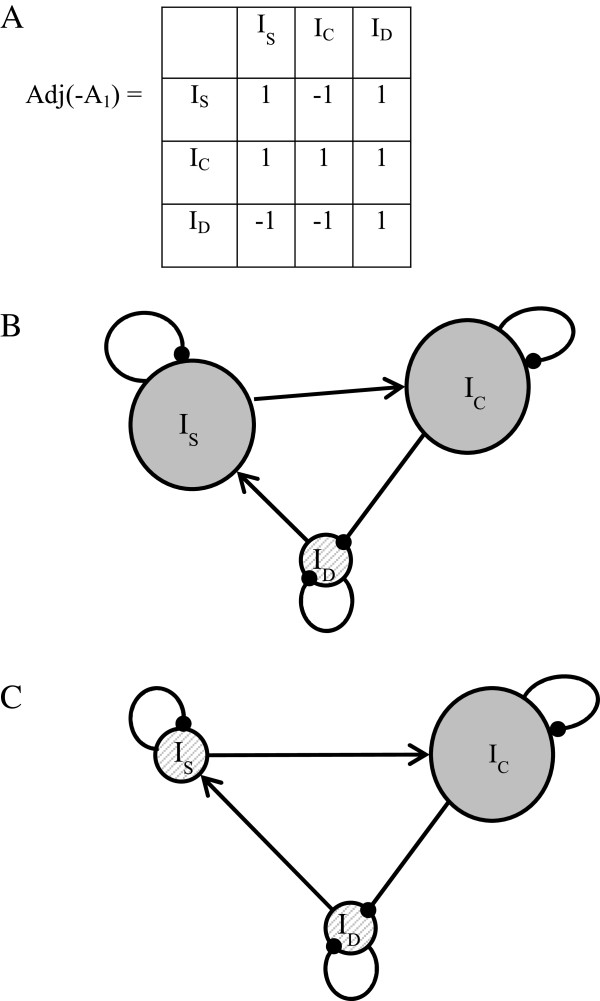
**Study of the dynamic of the generic system.** I_S_: Intensity of event-based surveillance. I_C_: Intensity of control. I_D_: Detected intensity of the disease. **A**: Matrix adjoint to the generic system. Perturbations are read in the columns and the variables’ responses to perturbations are read in the corresponding rows of the matrix Adj(−A_1_). **B** and **C**: Directed graphs representing the response of the generic system to a positive perturbation on I_S_**(B)** and I_C_**(C)**. Greyed-out and enlarged circle: increasing variable. Hashed-out and reduced circle: decreasing variable.

1) In the case of a country affected by an infectious animal disease, the simplest model that can describe its surveillance and intervention activities is a generic one with three intensity variables, called model 3I. The intensity of the disease (variable I_D_) represents the level of disease occurrence detected by the surveillance system. I_S_ and I_C_ represent the intensity of surveillance and intervention, respectively, in other words, the extent of the financial, human and material resources deployed for the surveillance and control of the disease. We excluded systems designed for the early detection of outbreaks, where I_D_ and I_C_ are null.

2) We assumed that the 3I system is close to a state of equilibrium at every moment. This implies that the disease intensity (variable I_D_) is close to or oscillates around the equilibrium: this corresponds to an endemic disease situation. Thus, the 3I model is further restricted to the case when the disease is present and endemic. We also considered the 3I model interactions being continuous, monotone and of the same order of magnitude.

3) We defined the interactions of our generic model as follows: first, I_D_ has a positive influence on I_S_. This is only true if I_S_ is restricted to event-based surveillance (i.e. surveillance based on the reporting of the disease cases spontaneously or by key stakeholders of the system). On the contrary, active surveillance can stay at a constant intensity level if the disease intensity increases (e.g. systematic surveillance). Consequently, I_S_ was further restricted to the intensity of event-based surveillance. We also excluded the study of the period of eradication because in this case the interaction I_D_-I_S_ would be negative. Indeed, if the prevalence of the disease approaches zero, surveillance needs to be increased to detect the last, sparser, remaining cases. I_S_ has a positive action on I_C_ (we assumed in our model that if more cases are detected, more cases will be later controlled). Finally, I_C_ has the effect of reducing I_D_ (we supposed that the deployed control measures effectively reduce the level of the disease). I_S_ and I_C_ are self-limiting (each having a negative action on themselves), as they are subject to limited financial, material and human resources. I_D_ is also self-limiting as the disease is endemic (if I_D_ increases, I_D_ later decreases and returns to its level of equilibrium). Finally, we excluded the emergence phase where the interaction I_D_–I_D_ is positive. The directed graph representing the generic system is presented in Figure 1A.

4) We converted this directed graph into an interaction matrix A_1_ represented in Figure 1B.

5) The 3I system meets both the Hurwitz stability criteria, and can therefore be considered stable.

6) The adjoint matrix Adj(-A_1_) describes the behavior of the 3I system after a positive perturbation (i.e. increase) of the variables in the columns headers (Figure 2A). The effect of a positive perturbation to I_S_ and I_C_ (read in the first and second columns of Adj(-A_1_) ) are also graphically displayed in the Figures 2B and 2C. By creating a positive perturbation on I_S_ or I_C_, the model shows a negative influence on I_D_, therefore a reduction in the detected disease level. However, by increasing I_C_ (Figure 2B), we can note that I_S_ also diminishes; the intensity of surveillance is reduced.

7) The predictions from the adjoint matrix are certain because all the coefficients of the matrix W_1_ associated with A_1_ have a value of 1.

Finally, the application of loop analysis to the generic model restricted the applicability of the method to event-based surveillance systems of endemic diseases, excluding the emergence and eradication phases. The HPAI H5N1 mitigation system in Cambodia fulfils these requirements: HPAI H5N1can be considered as endemic in Cambodia, as it has been present since 2004 according to the OIE, circulating mostly at a low level among humans and domestic poultry [25]. Moreover, this mitigation system is mainly based on event-based surveillance [26]. Thus, we used the HPAI H5N1 mitigation system in Cambodia as an example to study the application of loop analysis to a real mitigation system. To apply loop analysis to this particular case, we considered each variable of the generic model and redefined it according to this specific context. For example, the intensity of the surveillance was modeled by combining several interacting variables representing the intensity of surveillance performed by each of the stakeholders of the surveillance system, including the farmers themselves. Thus to elaborate the specific model, we broke down the generic one into one with more variables and more interactions. Building this more complex model therefore required the prior understanding of the HPAI H5N1 mitigation system in Cambodia. By improving our knowledge of the system, we also improved our ability to build a more precise model, that is to say, one with more specific interactions.

## Results

We present in this section the application of the 7-steps loop analysis approach to the HPAI H5N1 mitigation system in Cambodia. The first part of the section focuses on the steps 1 to 4, and the second part on the steps 5 to 7.

### Description of the HPAI H5N1 mitigation system in Cambodia

Our knowledge of the HPAI H5N1 mitigation system in Cambodia was based on bibliographic data [26,27] and a field study conducted in April 2012 in the Cambodian provinces of Kompong Cham and Takeo. In the course of this study, we interviewed key stakeholders of the system to collate different points of view on the functioning of the system. Key stakeholders consisted of 21 farmers, 21 designated Village Animal Health Workers (VAHWs), 6 district veterinarians, 3 province-level veterinarians and 3 NaVRI (National Veterinary Research Institute) personnel. The VAHWs are village volunteers, usually one per village, who have been trained to recognize, treat and report certain animal diseases to the health authorities, in particular cases of HPAI H5N1. In our selection of local stakeholders we targeted active VAHWs working in zones affected by previous HPAI H5N1 outbreaks, whose names were put forward by the district veterinarians. We then interviewed farmers in the village of each selected VAHW. Backyard farming makes up the majority of poultry production in Cambodia [28], and it is reasonable to assume that the behavior of poultry farmers is generally homogeneous throughout the country. Thus, the selected sample gave us a satisfactory representation of the role of local stakeholders within the network, in particular of their surveillance and control activities, and interactions with other stakeholders.

1) We called I_D_ the intensity of the HPAI H5N1 in Cambodia. Further to the introduction of this disease on the territory in 2004, the Cambodian government, with the support of various international organizations, has put in place a system for the surveillance and control of the disease [26]. This system is principally based on event-based surveillance, organized into three levels. The local level consists of farmers and VAHWs, who monitor and control HPAI H5N1. We called I_Sl_ the intensity of farmers and VAHWs surveillance, measured as the extent of the human, financial and material resources these stakeholders use to monitor livestock (principally by observation), and to report cases to the intermediate level if appropriate. Similarly, I_Cl_ represents all the financial, material and human resources implicated in the control activities by the local stakeholders. It includes disinfecting premises, or separating and treating diseased animals. For farmers and VAHWs, I_D_ represents the number of dead or diseased birds.

The intermediate level, consisting of district and province-level veterinarians, also plays a role in event-based surveillance, by passing on information from the local level to the national level. The I_Si_ variable is the extent of the material, human and financial resources required by the district and province veterinarians for the surveillance of the sanitary situation in farms, and its reporting if required. The intensity of the surveillance by the CDC (Centre for Disease Control and Prevention), working under the aegis of the Ministry of Health, can also be integrated into the variable I_Si_. Indeed, this intermediate actor transfers information acquired via its own hotline, from local stakeholders to the NaVRI. In addition, the NaVRI conducts event-based surveillance of suspected cases of HPAI H5N1 at national level. It receives calls to the hotline, and if HPAI H5N1 is suspected, it intensifies its surveillance by collecting and analyzing samples. The intensity of this surveillance, labeled I_Sn_, represents the extent of human, financial and material resources deployed by the NaVRI to evaluate the disease’s level of occurrence. If HPAI H5N1 is confirmed, the NaVRI implements control measures. It organizes in particular the selective and uncompensated slaughter of animals that test positive for HPAI H5N1, as well as the disinfection of the area and the prevention of animal movements. I_Cn_ corresponds to the human, material and financial resources put in place for the slaughtering, disinfection and blocking the transport of animals. Intermediate stakeholders take few decisions relating to control. Rather, they are more involved in the application of national control measures, as decided by the NaVRI. We therefore chose to include the intensity of the control by intermediate stakeholders in the variable I_Cn_. The NaVRI also carries out active surveillance of HPAI H5N1 in live poultry markets and sentinel free-range duck farms. This surveillance is conducted by scheduled spot checks, and its intensity is independent from the epidemiological dynamics of the disease. We were therefore unable to integrate it into our model. Indeed, if a variable describing the intensity of active surveillance was included, it would be isolated and have no interaction with the other variables. However, this is of no consequence for the study of the system’s dynamics as such. A summary table of the stakeholders involved at different levels, and the corresponding variables is presented in the Table 1.

**Table 1:** Summary table of the stakeholders involved in the HPAI H5N1 mitigation system in Cambodia and the corresponding variables included in the model

**Level**	**Stakeholders**	**Variables: surveillance intensity**	**Variables: control intensity**
National	NaVRI	I_Sn_	I_Cn_
Intermediate	District and provincial-level veterinarians, CDC	I_Si_	I_Cn_
Local	Farmers, VAHWs	I_Sl_	I_Cl_

The surveillance (respectively control) intensity variables represent the extent of the human, financial and material resources deployed by the stakeholders involved in the HPAI H5N1 surveillance (respectively control) in Cambodia.

To adapt the generic model to the context of the mitigation system of HPAI H5N1 in Cambodia, we broke down the variable I_S_ into three variables (I_Sl_, I_Si_ and I_Sn_), corresponding to the different surveillance levels. At the same time, we broke down the variable I_C_ into two new variables (I_Cl_ and I_Cn_), to reflect the two main levels of control. This breakdown was discussed and validated with two members of the NaVRI and one of the authors of this article, an expert on the surveillance network for HPAI H5N1 in Cambodia.

**Figure 3:**
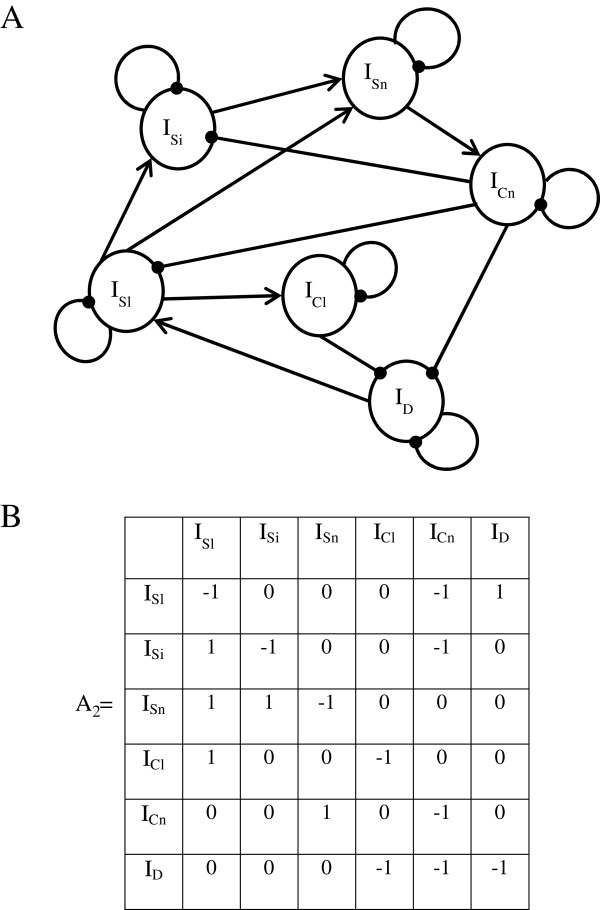
**Representation of the HPAI H5N1 mitigation system in Cambodia.** I_Sl_: Intensity of surveillance by local stakeholders (farmers, VAHWs), I_Si_: Intensity of surveillance by intermediate stakeholders (district and province-level veterinarians, CDC), I_Sn_: Intensity of surveillance by national-level stakeholders (NaVRI), I_Cl_: Intensity of control by local stakeholders, I_Cn_: Intensity of control by intermediate and national stakeholders, I_D_: Detected occurrence level of the disease (number of dead or diseased birds). **A**: Directed graph. Pointed end arrows: positive interaction. Circular end arrows: negative interaction. **B**: Matrix A_2_ of interactions. **A** and **B** are two equivalent representations of the HPAI H5N1 mitigation system in Cambodia. To get the interaction matrix A_2_ from the directed graph, only the direction and the sign of the interactions are taken into account, and not their amplitude.

2) We considered here that HPAI H5N1 is endemic in Cambodia. This hypothesis seems acceptable, as HPAI H5N1 has been present in Cambodia since 2004 according to the OIE, circulating mostly at a low level among humans and domestic poultry [25]. We also decided to represent here the national scale, meaning that I_Sl_ and I_Cl_ include (aggregate) the intensities of surveillance and control of all farmers and VAHWs in Cambodia. Thus we considered that the intensity of national surveillance and control was of the same order of magnitude as the aggregated intensities of surveillance and control at local level.

3) We then described the interactions between the system’s variables. The link between I_D_ and I_Sl_ is positive: the farmers and VAHWs intensify surveillance when they observe affected birds; they monitor their flocks more frequently and cautiously, and are more diligent about reporting cases. We supposed that if the intensity of the local surveillance increases, the intensity of the local control increases (positive I_Sl_-I_Cl_ link). Assuming that local control is effective in reducing the incidence of the disease, I_Cl_ in turn then has a negative influence on I_D_ (negative I_Cl_- I_D_ link). I_Sl_ influences I_Si_ positively (positive I_Sl_-I_Si_ link). Indeed, the resource investment increases when an intermediate-level veterinarian is contacted by a farmer or VAHW, for example, if the veterinarian then makes a field visit to collect samples. I_Sn_ increases when the intensity of the surveillance by local (positive I_Sl_-I_Sn_ link) and intermediary stakeholders (positive I_Si_-I_Sn_ link) increases, due to hotline reports. We chose to examine a scenario where a case of HPAI H5N1 is confirmed. The I_Sn_-I_Cn_ link is therefore positive – without confirmation, there is no such link. I_Cn_ has a negative action on I_D_: we assumed that the control at national level effectively reduces the occurrence of the disease. I_Cn_ also has a negative influence on I_Sl_ and I_Si_ (negative I_Cn_-I_Sl_ and I_Cn_-I_Si_ links). Indeed, increasing the number of animals slaughtered without compensation reduces the reporting of cases by local and intermediate stakeholders, who are affected by these measures [27]. Figure 3A shows a proposed representation of the directed graph representing this system.

4) This directed graph was then converted into the interaction matrix A_2_ showed in Figure 3B.

Thus, loop analysis offered a new representation of the HPAI H5N1 mitigation system in Cambodia. Representing the components of the system and the interactions between them improved our understanding of the system’s structure and functioning. It was especially interesting to understand how surveillance and control are connected, as this is usually unclear with classical representations of this system (hierarchical organigramme). Breaking down the generic model to build a more complex model allows for keeping surveillance components and control components together, making the link between surveillance and control clear.

### Study of the dynamic of the HPAI H5N1 mitigation system in Cambodia

This section focuses on the steps 5 to 7 of the loop analysis approach applied to the HPAI H5N1 mitigation system in Cambodia.

5) The system for the surveillance and control of HPAI H5N1 in Cambodia is considered stable in that it meets both Hurwitz stability criteria.

6) Thus, we studied the predictability of this system by calculating its conjugate transpose presented in Table 2.

**Table 2:** Matrix adjoint to the HPAI H5N1 mitigation system in Cambodia

		**I**_**Sl**_	**I**_**Si**_	**I**_**Sn**_	**I**_**Cl**_	**I**_**Cn**_	**I**_**D**_
	I_Sl_	2	−2	−2	−2	−2	2
	I_Si_	0	4	−4	0	−4	0
Adj (−A_2_) =	I_Sn_	2	2	2	−2	−6	2
	I_Cl_	2	−2	−2	6	−2	2
	I_Cn_	2	2	2	−2	2	2
	I_D_	−4	0	0	−4	0	4

I_Sl_: Intensity of surveillance by local stakeholders (farmers, VAHWs), I_Si_: Intensity of surveillance by intermediate stakeholders (district and province-level veterinarians, CDC), I_Sn_: Intensity of surveillance by national-level stakeholders (NaVRI), I_Cl_: Intensity of control by local stakeholders, I_Cn_: Intensity of control by national-level and intermediate stakeholders, I_D_: Detected level of disease occurrence (number of dead or diseased birds).

Perturbations are read in the columns and the variables’ responses to perturbations are read in the corresponding rows of the matrix Adj(−A_2_).

**Figure 4:**
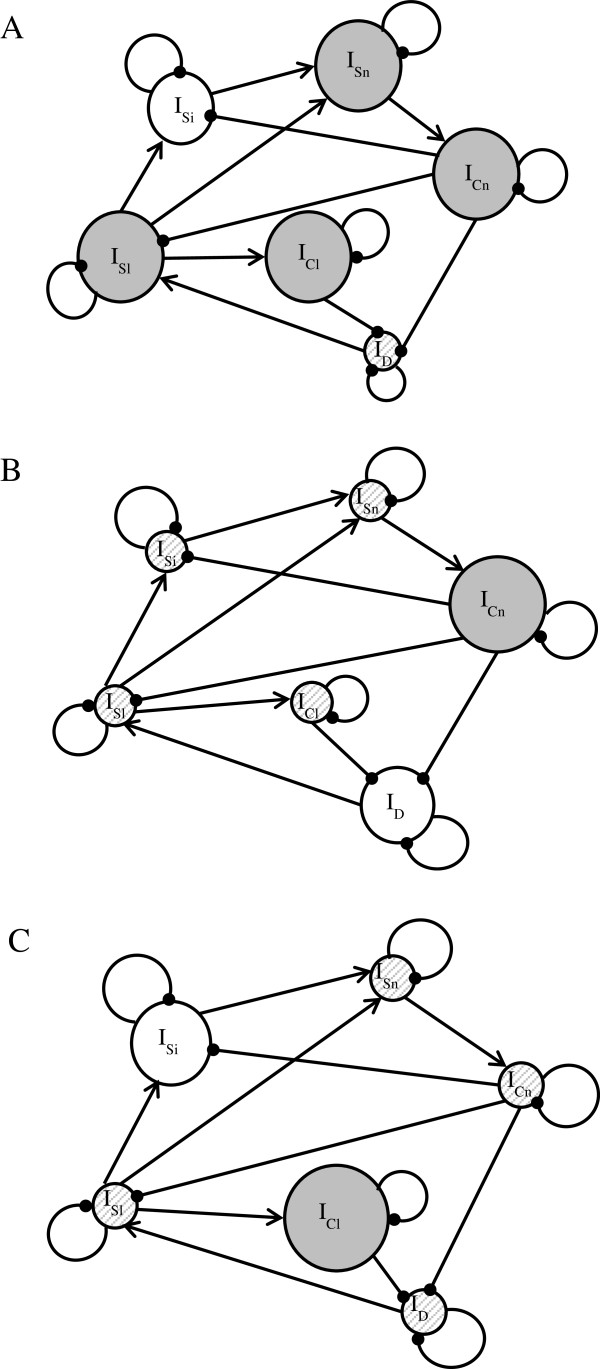
**Study of the dynamic of the HPAI H5N1 mitigation system in Cambodia.** Impact of a positive perturbation on I_Sn_**(A)**, I_Cn_**(B)** and I_Cl_**(C)**. I_Sl_: Intensity of the surveillance by local stakeholders (farmers, VAHWs), I_Si_: Intensity of the surveillance by intermediate stakeholders (district and province-level veterinarians, CDC), I_Sn_: Intensity of the surveillance by national-level stakeholders (NaVRI), I_Cl_: Intensity of the control by local stakeholders, I_Cn_: Intensity of the control by national-level and intermediary stakeholders, I_D_: Detected level of incidence of the disease (number of dead or diseased birds). Greyed-out and enlarged circle: increasing variable. Hashed-out and reduced circle: decreasing variable. White circle: ambiguous direction of change of the variable (impossible to conclude if it increases or decreases).

We elected to focus on the positive perturbations on I_Sl_, I_Cl_ and I_Cn_ (read in the first, fourth and fifth columns of the matrix Adj(−A_2_)). To facilitate the reader’s understanding, the behavior of the system in these three cases was represented in three distinct directed graphs (Figure 4).

The results show that a positive perturbation on I_Sl_ (Figure 4A) increases the intensity of surveillance (except for I_Si_, which is ambiguous) and of the control across the different stakeholders, and reduces the intensity of the disease. Furthermore, a positive perturbation of the intensity of national control (Figure 4B) reduces the intensity of surveillance at all levels, and that of control at local level. On other hand, the impact on I_D_ is ambiguous: the model does not indicate whether this national-level control is effective in reducing the level of the disease. Finally, results indicate that increasing the intensity of local control (see Figure 4C for the positive impact on I_Cl_) reduces I_D_ unambiguously while lowering the intensity of local and national-level surveillance (with ambiguity for I_Si_).

7) We calculated the matrix W_2_ associated to the system; it is presented in Table 3. We note that six predictions out of the 36 are completely ambiguous, which indicates that only a feeble level of confidence is attributed to them. This is linked to the presence of feedback loops of opposite signs within the system [19], which reduces the net number of complementary feedbacks.

**Table 3:** **Matrix W**_2_**of the predictions relating to HPAI H5N1 mitigation system in Cambodia**

		**I**_**Sl**_	**I**_**Si**_	**I**_**Sn**_	**I**_**Cl**_	**I**_**Cn**_	**I**_**D**_
	I_Sl_	1	1	1	1	1	1
	I_Si_	0	1	1	0	1	0
W_2_ =	I_Sn_	1	1	1	1	1	1
	I_Cl_	1	1	1	1	1	1
	I_Cn_	1	1	1	1	1	1
	I_D_	1	0	0	1	0	1

I_Sl_: Intensity of surveillance by local stakeholders (farmers, VAHWs), I_Si_: Intensity of surveillance by intermediate stakeholders (district and province-level veterinarians, CDC), I_Sn_: Intensity of surveillance by national-level stakeholders (NaVRI), I_Cl_: Intensity of control by local stakeholders, I_Cn_: Intensity of control by national-level and intermediate stakeholders, I_D_: Detected level of disease occurrence (number of dead or diseased birds).

The matrix coefficients represent the level of confidence to be attributed to the predictions, ranging from unambiguous at level w_ij_ = 1 (certain prediction), to totally ambiguous at w_ij_ = 0 (very poor confidence in the prediction). A value of w_ij_ = 0.5 is generally accepted as a validation threshold.

## Discussion

### Loop analysis conditions for application and limits

We have described the principle of the loop analysis method, and illustrated its application, first to a minimal, generic model (3I), then to a specific model of the HPAI H5N1 mitigation system in Cambodia. The loop analysis method imposes certain restraints which limit its applicability to the context of disease mitigation systems. The study of the 3I model showed that loop analysis can be applied to any disease subject to event-based surveillance and control. However, the application is limited to endemic diseases; this corresponds to the epidemiological phase when the system is close to a state of equilibrium, which means that the intensities of the disease, its surveillance and its control vary little over time. We can accept this hypothesis for a disease that has been endemic for several years, as in the case of HPAI H5N1 in Cambodia. On contrary, the method cannot be applied to transition phases such as emergence and eradication phases.

The generic and the HPAI H5N1 models are, furthermore, based on the assumption that control measures are effective in reducing the occurrence of the disease, which is debatable. At national level, only cases detected by the system are controlled, effectively making the effectiveness of such controls dependent on the sensitivity of the surveillance system. The field implementation of local control measures can also be questioned, for example with some farmers selling their diseased animals, thereby contributing to the spread of HPAI H5N1 [29].

The method is also based on the assumption that the rate of variation is of the same order of magnitude for all variables. To consider this hypothesis in our model for Cambodia, we assumed that the intensity of national surveillance and control is of the same order of magnitude as the aggregated intensities of surveillance and control at local level. However, further works would be needed to confirm this hypothesis. Another limitation of the model is that some interactions between the compartments of the system are subject to threshold effects. For example, a farmer may tend to wait until he has observed a certain number of dead animals before reporting the case to health authorities, even for suspected bird flu [30,31]. Loop analysis does not consider these thresholds, as it assumes a continuous interaction between variables. Working at a national level, i.e. aggregating all the Cambodian farmers’ surveillance intensities, we can assume that the directed interaction always exists. However, at an individual level, this assumption is not borne out: below the farmers’ reporting threshold the links I_Sl_-I_Si_ and I_Sl_-I_Sn_ do not exist. A new model should therefore be built eliminating these links for the individual level.

Finally, loop analysis does not allow for the weighting of the strength of interactions between the variables. Nonetheless, these strengths can be assumed to vary within our model for Cambodia. For example, the link I_Sl_–I_Cl_ is stronger than I_Sl_-I_Si_. Indeed, all farmers attempt to put in place their own controls when they observe dead or diseased animals, while only some report the cases to veterinary authorities, among other reasons because their trust in these institutions is limited [32]. However, the weighting of interactions can be integrated into our model by the use of the Fuzzy Cognitive Maps method, a semi-quantitative modeling tool for dynamic systems which would allow the refining of the models proposed by loop analysis [33].

The progression from the 3I model to a more precise model was carried out by specifying the variables and interactions laid out by the minimal model. This method requires only few data and is therefore easily adapted to the context of developing countries. We applied it to the case of surveillance and control of HPAI H5N1 in Cambodia, which led us to elaborate a model with six variables. The construction of the model improved our understanding of the system, especially because this model highlights the link between surveillance and control components, which is usually unclear in traditional representations of this system (hierarchical organigrammes). We feel that this breakdown method can be applied to any system for the surveillance and control of an endemic disease. However, the single example of Cambodia is not sufficient to demonstrate the method’s generality, i.e. the possibility to apply it to other mitigation systems. We are currently conducting further field work to this end in Thailand and Laos.

### Interpretation of the mitigation system’s behavior: the study of the system’s efficiency

As the 3I model and the model for Cambodia are stable, we were able to study the responses of the systems to perturbations of their compartments. We propose here to attribute a financial, human and material cost to the intensity of surveillance and control. The hypothesis of a positive correlation between cost and intensity of surveillance and control seems acceptable from a qualitative point of view. We can then interpret a positive perturbation of a surveillance or control compartment as an investment made in this compartment in terms of financial, human and material resources deployed to achieve the surveillance or the control of the disease. Starting from there, we can study the effect of an investment in a certain compartment on the costs of surveillance or control and on the disease occurrence level. We considered here that the investments causing both a reduction of the surveillance and control associated costs and the disease occurrence level a priori optimize the system’s efficiency, that is to say its effectiveness in reducing the occurrence of the disease by optimizing the financial, human and material resources deployed. However, we should keep in mind that optimizing the system’s efficiency does not necessary mean reducing the surveillance and control activities costs to reduce the disease occurrence level. It could also consist in making better use of the resources available, at a constant cost level.

Nevertheless, we think that this qualitative modeling approach is an interesting first step to study the efficiency of surveillance and control systems in animal health. Indeed, it is very hard to quantify the effect of an investment, especially when we work with partial or imprecise data. Additionally, the application of policies for the surveillance and control of diseases relies on human behavior, which is also hard to measure or quantify [34]. The loop analysis being a qualitative method, we cannot, by definition, quantify the decrease or increase in efficiency. However, we can calculate its trend according to an investment choice. This is the result of the analysis of the perturbations, which allow us to rank various situations according to the perturbation’s effect on the system’s efficiency.

The results of the analysis by the 3I and Cambodia-specific models show a dynamic common to both systems: investing in control measures corresponds to a reduction in the occurrence of the disease without increasing the intensity of surveillance. On the contrary, an investment in surveillance requires an additional increase in control to achieve a reduction in the occurrence of the disease. Investing in control rather than surveillance a priori yields a better improvement in the system’s efficiency.

In the case of the model for Cambodia, the model shows that investments in local surveillance (for example working to improve farmers’ trust in veterinary services, and encouraging them to report observed cases of disease), may contribute to reduce the intensity of the disease (Figure 4A). This reduction requires increased intensity of both surveillance and control of all stakeholders within the system, meaning at national level and all local stakeholders in Cambodia. (There is some ambiguity regarding intermediate stakeholders, we are unable to conclude whether the intensity of their surveillance increases or decreases). Even if we cannot quantify the different costs associated with the rises in intensity, the model predicts that this situation will be characterized by an overall increase in the cost of surveillance and control relative to the former equilibrium state. Thus, results indicate that the occurrence of the disease would be reduced but with an elevated cost, so the efficiency of the system would decrease in this case.

Conversely, results suggest that investing in control measures carried out at the national level can reduce the intensity of surveillance (Figure 4B), thereby limiting the costs incurred by this surveillance. However, the effect on I_D_ is ambiguous in this scenario, and we cannot therefore draw any conclusions regarding the effectiveness of this type of investment to reduce the occurrence of the disease. This ambiguity can be explained by the negative influence of uncompensated slaughter on the reporting of cases by local and intermediate stakeholders. If we construct an identical system but eliminate the negative links I_Cn_-I_Sl_ and I_Cn_-I_Si_, we notice that an investment in control at national level allows for the occurrence of the disease to be unambiguously reduced. Thus, by working on the acceptability of national control measures, the effectiveness of the system to control the disease can be improved [35]. The type of investment presented in Figure 4B therefore allows for the reduction of surveillance costs, but its effectiveness in reducing disease occurrence is ambiguous, which means the efficiency of the system is ambiguous in this case.

Finally, a positive perturbation on the intensity of local control (Figure 4C) reduces unambiguously the intensity of the disease, while reducing the intensity of the surveillance and control at national level. Local control has two distinct advantages: on the one hand it does not negatively influence the intensity of surveillance, and on the other hand it does not require laboratory confirmation of HPAI H5N1 cases. The case in Figure 4C therefore represents a situation which should in principle be less costly than the one in Figure 4A (by reduction in the intensity of surveillance and control), and more effective that the one in Figure 4B in reducing the occurrence of the disease. The scenario represented in Figure 4C therefore provides for the optimization of the system’s efficiency.

We adopted here the perspective of a decision-maker, with a good overall visibility of the system, to ask ourselves what scenario would optimize its efficiency. However, it should be noted that depending on the approach, a single given system can be represented by one of several loop analysis models. Indeed, the validity of any particular model structure depends only on a compromise between different points of view, and the interpretation of impacts depends on the question at hand. Our qualitative approach does not allow the quantification of the investment necessary to optimize the functioning of the system. However, by understanding the system’s dynamics, the model allows different situations to be ordered hierarchically, and the most favorable configuration for optimizing the system’s efficiency to be identified. This analysis can therefore be considered to be a precursor to a large-scale quantitative project, and allows for the targeting of data to collect. Thus, in the case of the model for Cambodia, it could be interesting to evaluate quantitatively and precisely the costs of reinforcing the control of HPAI H5N1 by local stakeholders. Indeed, according to the results of the model, the scenario in Figure 4C allows for the best optimization of the system’s efficiency. This underlines the importance of training local stakeholders, specifically to control avian diseases at their level, and also supports the conclusions of other studies that have shown the importance of involving local stakeholders in the improvement of the efficiency of systems of surveillance and control of HPAI H5N1 [36], and animal diseases more widely [37].

## Conclusion

Loop analysis can be adapted to the context of animal disease mitigation systems. This only requires defining the system’s components and the sign of their interactions, but not their value. This is the main strength of the method (it is easy to design), but also its main limit, as we can only say whether the variables increase or decrease after a perturbation, but not how much or how fast. That is why loop analysis should not be used as a single method but in combination with quantitative ones.

Starting from a simple and generic model, it is possible to develop a model suitable to a specific context by breaking down the variables into sub-variables. In this way we were able to obtain a model with six variables organized into three levels, describing the mitigation system of HPAI H5N1 in Cambodia. This improved our understanding of the system’s organization, especially highlighting the link between surveillance and control. Loop analysis also allows for the impacts on the system’s different compartments to be studied. We chose to interpret these impacts in terms of financial, human and material investments. The results show, among others, that investing in the compartment corresponding to local-level control represents the most favorable scenario for optimizing the efficiency of the system.

We think the loop analysis could also be used as a participatory epidemiological tool. For example, it would allow different stakeholders of the network to build a common representation of their system, and to study its dynamic through various perturbations. This type of approach is already applied in the field of ecology, for example in teaching the carbon cycle and the greenhouse effect [38]. The value of this method lies in its simplicity and flexibility, as these models are fast to build, analyze and modify, providing a good aid for study and debate. A systemic approach to a disease mitigation system can allow its complexity to be reduced and its dynamics to be better understood, thereby stimulating new ways of conceptualizing these systems. This could also raise local stakeholders’ awareness of these dynamics, thereby giving them a better understanding of the consequences of their actions.

## Abbreviations

CDC: Centre for Disease Control and Prevention; HPAI: Highly Pathogenic Avian Influenza; NaVRI: National Veterinary Research Institute; VAHW: Village Animal Health Worker.

## Competing interests

The authors declare that they have no competing interests.

## Authors’ contributions

LC and RD drafted the original manuscript. FG and SH supported the fieldwork organization in Cambodia and took part in the models discussion. All authors commented on successive drafts of the manuscripts and read and approved the final manuscript.
